# Diplodia tip blight pathogen’s virulence empowered through host switch

**DOI:** 10.3389/ffunb.2022.939007

**Published:** 2022-07-28

**Authors:** Kathrin Blumenstein, Johanna Bußkamp, Gitta Jutta Langer, Eeva Terhonen

**Affiliations:** ^1^ Forest Pathology Research Group, Department of Forest Botany and Tree Physiology, Faculty of Forest Sciences and Forest Ecology, Georg-August-University Göttingen, Göttingen, Germany; ^2^ Chair of Pathology of Trees, Institute of Forestry, Faculty of Environment and Natural Resources, University of Freiburg, Freiburg, Germany; ^3^ Section Mycology and Complex Diseases, Department of Forest Protection, Northwest German Forest Research Institute, Göttingen, Germany; ^4^ Natural Resources Institute Finland (Luke), Forest Health and Biodiversity, Helsinki, Finland

**Keywords:** climate change, conifers, *Diplodia sapinea*, host-passage, *Pinus sylvestris*, *Sphaeropsis sapinea*, symptomatic and asymptomatic infections, water-stress

## Abstract

Increased drought combined with emerging pathogens poses an increased threat to forest health. This is attributable to the unpredictable behaviour of forest pathosystems, which can favour fungal pathogens over the host under persistent drought stress conditions. *Diplodia sapinea* (≡ *Sphaeropsis sapinea*) is one of the most severe pathogens in Scots pine (*Pinus sylvestris*) causing Diplodia tip blight (conifer blight) under certain environmental conditions. Recently, the fungus has also been isolated from non-conifer hosts, indicating that it has a broader host range than previously known. In this study we compared the impact of different levels of water availability on necrosis length caused by *D. sapinea* strains isolated as endophytes (eight strains isolated from asymptomatic Scots pine) and pathogens (five strains isolated from symptomatic Scots pine) and five strains isolated from symptomatic non-pine hosts. For all strains the decreased water availability increased the necrosis length in Scots pine shoots. The isolates from non-pine hosts caused the most severe reactions under all water availabilities. The results of the study indicate the likelihood that effects of climatic changes such as drought will drive *D. sapinea* damage in Scots pine-dominated forests and increase mortality rates in affected trees. Further, the higher necrosis in the Scots pines caused by strains that had performed a host switch are concerning with regard to future scenarios thus increasing infection pressure on Scots pine from unknown sources.

## 1 Introduction

Scots pine (*Pinus sylvestris* L.), together with Norway spruce (*Picea abies* (L.) H. Karst.), forms the basis for raw materials of the forest sector in Europe. Scots pine is not only highly economically important but also is of ecological value in Europe, particularly in Northern and Central European countries. Further, Scots pine is considered tolerant of drought, although recent observations show that prolonged water and nutrient deficiencies will cause severe damage in the species (Gomez-Gallego et al., 2022). Much of this damage is due to the interaction between extended drought and the emerging pathogen *Diplodia sapinea* (Fr.) Fuckel (≡* Sphaeropsis sapinea* (Fr.) Dyko & B. Sutton*)* (Bußkamp, 2018; Blumenstein et al., 2021a; Blumenstein et al., 2021b) causing shoot blight in Scots pine. Climate change, exemplified by increased drought, poses a unique threat to European Scots pine forest health. *Diplodia sapinea* is present as an endophyte in the tissue and can become pathogenic, causing Diplodia tip blight, in drought-stressed trees. Because of rapid changes in climate and long tree generations, Scots pines in Europe may not be able to keep up with these adjustments, making them more susceptible to drought stress and *D. sapinea* invasion. The increase in outbreaks is already happening in South (Oliva et al., 2020) and Central Europe (Bußkamp, 2018; Adamson et al., 2021; Blumenstein et al., 2021b) and increasing gradually in North Europe (Brodde et al., 2019; Terhonen, 2022). In Germany, *D. sapinea* is widespread in healthy and diseased trees (Bußkamp, 2018; Blumenstein et al., 2021a; Bußkamp et al., 2021).

The correct name of this anamorphic Botryosphaeriaceae is under discussion, but the current name after Index Fungorum (accessed 31.5.2022) is still *S. sapinea*. Within its life cycle, *D. sapinea* has different trophic modes: asymptomatic endophyte (Terhonen et al., 2021), opportunistic pathogen and/or saprotroph. In combination with abiotic stressors, such as drought (Blumenstein et al., 2021b), hail (Oliva et al., 2020), or extreme temperatures (Bußkamp, 2018), *D. sapinea* rapidly becomes pathogenic, leading to sudden disease outbreaks in mature trees (Brodde et al., 2019; Oliva et al., 2020; Blumenstein et al., 2021a) and regenerating saplings (Larsson et al., 2020). *Diplodia sapinea* reproduces mainly asexually, resulting in small genetic differences between subpopulations at the regional level (Adamson et al., 2021). Nevertheless, differences in virulence and pathogenicity between isolates from the same subpopulation have been found in Germany (Bußkamp et al., 2021; Langer and Bußkamp, 2021). *Diplodia sapinea* has multiple tree hosts, preferring coniferous species. Recently, *D. sapinea* has been isolated from non-coniferous host trees, such as Black alder (*Alnus glutinosa* (L.) Gaertn.) (Bußkamp et al., 2021) and European beech (*Fagus sylvatica* L.) (Langer and Bußkamp, 2021). Strains from these non-conifer hosts can be extremely aggressive in Scots pine (Bußkamp et al., 2021). The wide potential host range of *D. sapinea* (Langer and Bußkamp, 2021), high isolate aggressiveness (Bußkamp et al., 2021), increasing disease severity under drought stress (Blumenstein et al., 2021b) and its ability to accumulate as an endophyte (Blumenstein et al., 2021a; Terhonen et al., 2021) makes emerging *D. sapinea* a high-risk pine pathogen in Europe (Brodde et al., 2019; Oliva et al., 2020; Bußkamp et al., 2021; Terhonen et al., 2021). To examine the increased adaptability and mobility of this latent pathogen (compared to its hosts), we need to determine the specific virulence and pathogenicity of known subpopulations in different climates. To learn more about the detailed epidemiology of *D. sapinea*, herein, we provide essential basic information about the disease cycle of this fungus. The aim of this study was to show disease intensity as a function of abiotic stress, and to determine whether the pathogen changes its virulence after switching hosts.

## 2 Material and methods

### 2.1 Greenhouse study

#### 2.1.1 Fungal material

Twenty-one fungal isolates of *D. sapinea* (**Table 1**) were used in total, of which sixteen originated from *Pinus sylvestris* (**Table 1**). Eight strains were isolated from asymptomatic trees and considered to be endophytes (five strains: NW-FVA 2364, 2697, 2702, 2715, 5697 are the storage strains that were used in Bußkamp et al. (2021), two strains: NW-FVA 6073 and 6075 are re-isolates from strains NW-FVA 2715 and 2697 from the study Bußkamp et al. (2021), and three newly isolated strains: NW-FVA 6087, 6092, and 6292). Six strains were isolated from symptomatic pine material and considered to be pathogenic strains (NW-FVA 5305, 5306, 5329, 5747, and 5772 are the storage strains used in Bußkamp et al. (2021) and a re-isolate of NW-FVA 5747 (NW-FVA 6085) (from the study Bußkamp et al. (2021)). Five isolates originated from non-pine hosts: *A. glutinosa* (Bußkamp et al., 2021), *F. sylvatica* (Langer and Bußkamp, 2021), *Larix decidua* Mill., *P. abies* and *Pseudotsuga menziesii* (Mirbel) Franco (Bußkamp et al., 2021). Fungal twig isolates were obtained by surface sterilizing (1 min in 70% EtOH/5 min 4% NaOCl/1 min 70% EtOH) the plant material (conifer: twigs without needles) and cut into pieces (0.5 cm). Non-conifer isolates were isolated from surface sterilized necrotic tissues. These pieces were cultured on 1.5% Malt Yeast Peptone Agar (MYP, according to Bußkamp et al., 2020) at room temperature under a natural day-/night cycle. After 7 and 14 days the samples were checked for the presence of *D. sapinea*. Isolated strains were identified first visually based on morphology. Further DNA was extracted as described in Izumitsu et al. (2012). The ITS1-5.8S-ITS2 region of rDNA was amplified using primer pair ITS1-F (White et al., 1990) and ITS4 (Gardes and Bruns, 1993) as described in Terhonen et al. (2021). Sequences obtained were aligned and manually edited using MEGA5 software (Tamura et al., 2011), then used in BLAST searches of the GenBank databases (Altschul et al., 1997) to identify similar ITS sequences (similarity threshold of 98% was set for species-level identification). The ITS sequences of the fungal strains are deposited in GenBank (**Table 1**) and the strains are permanently stored in the NW-FVA strain collection, where they are freely available. Further, the species identity was confirmed by using the species-specific primers DiSapi-F (3´- CCCTTATATATCAAACTATGCTTTGT-5´) and Diplo-R (3´-TTACATAGAGGATTGCCTTCG-5´) as described in Adamson et al. (2021).

**Table 1:** List of *Diplodia sapinea* strains used in inoculations of the Scots pines, watering regimes and number of replicates (trees).

Treatment Number	Original host	Locality	Isolation year	Strain No.	NCBI accession number	Number of trees used for the treatment			
						25 % watering	60 % watering	100 % watering	Total
				NW-FVA		Drought stress	Median	Optimal	
1	*Pinus sylvestris* (asymptomatic, endophytic)	Hesse	2014	**2364**	MW529089	9	9	9	27
2		Saxony-Anhalt	2015	**2697**	MW529090	9	9	9	27
3		Re-isolate 2697	2021	6073*	ON127414	9	9	9	27
4		Bavaria	2015	**2702**	MW529091	9	9	9	27
5		Baden-Württemberg	2015	**2715**	MW529092	9	9	9	27
6		Re-isolate 2715	2021	6075*	MW529092	9	9	9	27
7		Lower Saxony	2020	**5697**	MW529100	9	9	9	27
8		Lower Saxony	2021	**6087**	ON127416	9	9	9	27
9		Lower Saxony	2021	**6092**	ON127417	9	9	9	27
10		Lower Saxony	2021	**6292**	ON127417	9	9	9	27
11	*Pinus sylvestris* (symptomatic, pathogenic)	Saxony-Anhalt	2019	**5305**	MW529094	9	9	9	27
12		Saxony-Anhalt	2019	**5306**	MW529095	9	9	9	27
13		Lower Saxony	2019	**5329**	MW529096	9	9	9	27
14		Saxony-Anhalt	2020	**5747**	MW529101	9	9	9	27
15		Re-isolate 5747	2021	6085*	ON127415	9	9	9	27
16		Saxony-Anhalt	2020	**5772**	MW529102	9	9	9	27
17	*Alnus glutinosa*	Mecklenburg-Western Pomerania	2020	**5654**	MW529099	9	9	9	27
18	*Fagus sylvatica*	Hesse	2019	**4932**	MN698984	9	9	9	27
19	*Larix decidua*	Lower Saxony	2016	**3626**	MW529093	9	9	9	27
20	*Picea abies*	Saxony-Anhalt	2020	**5355**	MW529098	9	9	9	27
21	*Pseudotsuga menziesii*	Hesse	2019	**5346**	MW529097	9	9	9	27
22		MYP		-		9	9	9	27
23		Non-treated		-		9	9	9	27
								Total	621

*Strains 6073, 6075 and 6085 were isolated from the infected Scots pines in the study by Bußkamp et al. (2021) as re-isolates. Numbers 6073 and 6075 originated from pines that were infected with the endophytic strain numbers 2697 and 2715, respectively, which are again included in this study. Number 6085 is the re-isolate from strain number 5747, also included in this study and originally a pathogenic strain. The strain’s original host, locality (the federal state in Germany), year isolated and the NCBI accession number.

#### 2.1.2 Plant material

**Figure 1:**
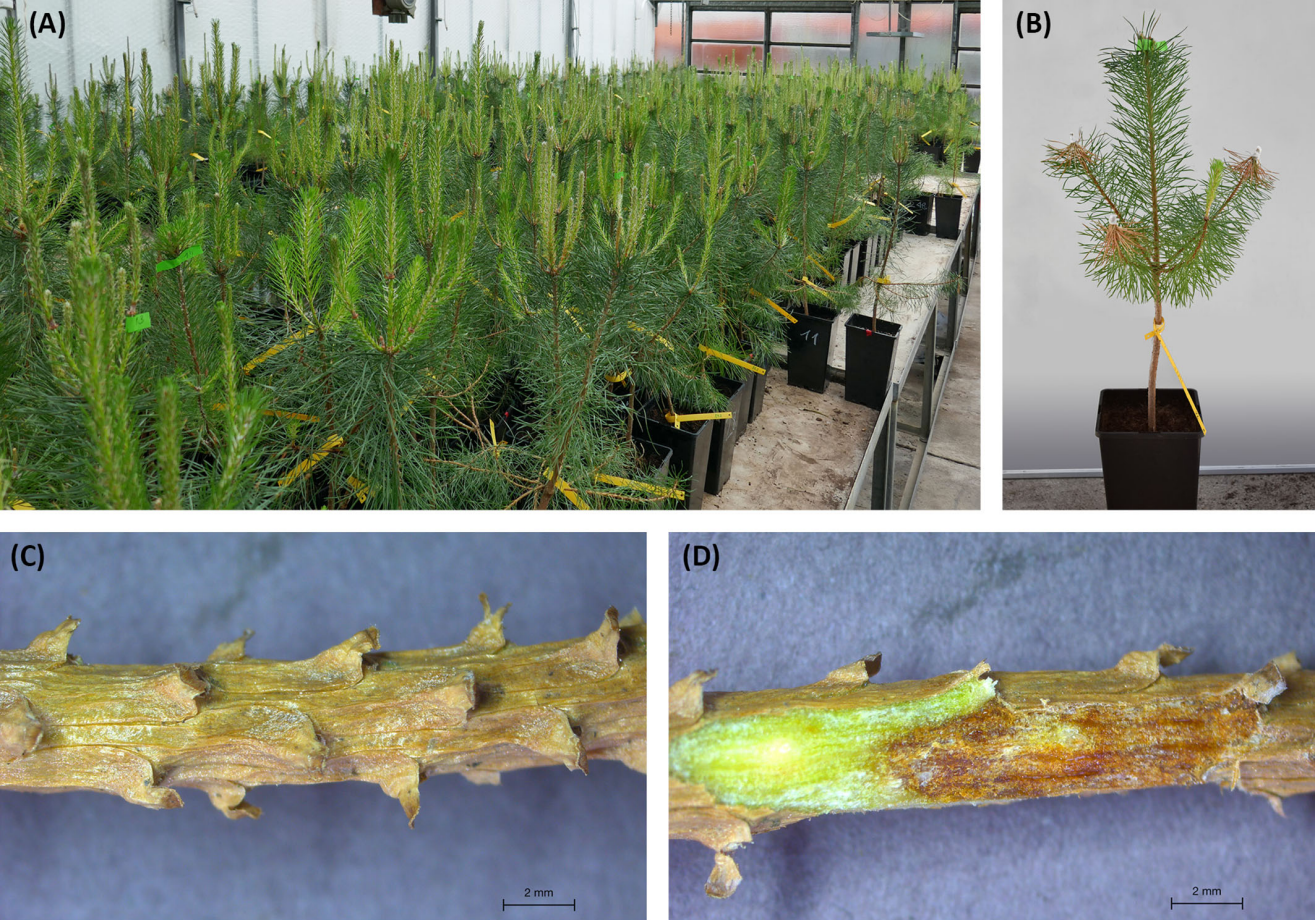
**(A)** Setup of Scots pines in the greenhouse; **(B)** symptoms due to *Diplodia sapinea* infections at the end of the experiment; **(C)** necrotic twig with bark; **(D)** necrotic twig with bark peeled off to measure the necrosis length.

To test the different virulence of *D. sapinea* strains under various levels of water availability, an *in vivo* infection study was performed at the greenhouses of the forest pathology group, University of Göttingen, Germany (51°33′28.4″ N 9°57′30.5″E). Altogether, 626 three-year-old *Pinus sylvestris* saplings were purchased from the Reinke tree nursery, Rellingen, Germany, originating from the provenance HKG 85103 Heide and Altmark, Germany and pre-planted in three litre pots (**Figure 1A**). The trees were arranged equidistant from each other on tables and watered to keep the peat sufficiently moist. The plants, which had three side-shoots, appeared to be very healthy at the beginning of the experiment, and no symptoms of Diplodia tip blight were visible. The tree height was measured at the beginning of the experiment and at the end. Mean temperature inside the greenhouse was 16.5°C (min 8°C and max 32°C).

#### 2.1.3 Pre-experiment detection of endophytes

*Diplodia sapinea* and other fungal endophytes are known to grow inside trees that do not show symptoms, even in young nursery trees. To make sure that the purchased trees were free from any known conifer pathogens, five of the saplings were randomly chosen and examined for any prior pathogenic infections before the start of the experiment. For this, branches, stems and needles were surface-sterilized and treated as described in Bußkamp et al. (2021). Frequency of isolated taxa, defined as portion of the number of isolated strains in relation to the total number of isolated filamentous strains, was calculated. Continuity of isolated taxa, defined as portion of the number of trees from which the fungus was isolated in relation to the total number of trees, was calculated.

#### 2.1.4 Experimental design of the greenhouse study

Four weeks prior to inoculation (April 2021), the saplings were grouped into three different water treatment groups: one group received the optimal amount of water (100%), one group 60% and one group 25% of the optimal amount. The saplings were randomly placed on tables. Before each watering, the soil moisture of 20 randomly chosen Scots pines per group (always the same 20) was measured using a DeltaT SM 150 Soil Moisture Kit (Delta-T Devices Ltd, UK). In May 2021, the Scots pine saplings were inoculated as described in Bußkamp et al. (2021) and (Blumenstein et al. 2021b; Figure 3). Briefly, each of the three side-shoots of the saplings were inoculated with a particular *D. sapinea* strain, mock-control or left untreated (**Table 1**), resulting in 27 saplings*3 inoculations = 81 inoculation points. The mycelium-agar plugs were covered with Parafilm^®^, no cross-contamination between the different strains and the trees was to be expected.

#### 2.1.5 Data collection and post-experiment detection of fungal species

The experiment was run for 23 days. The side-shoots were then detached with aseptic gardening scissors, and stored in paper bags at 4°C for immediate evaluation. Necrosis lengths were measured for each inoculated shoot with a ruler.

#### 2.1.6 Identification of re-isolated *D. sapinea* strains by vegetative compatibility tests

Re-isolations of the strains were performed for 66 inoculated trees (three trees per inoculated strain, six incubated tissue samples each, in total 396 tissue samples) and additionally three controls and three mock-controls were treated in the same way (36 tissue samples in total). To test the accordance of the re-isolated *D. sapinea* strains with original strains, vegetative compatibility tests were performed according to Burgess et al. (2009) and Milgroom and Cortesi (1999) with three replicates of the crossing of the re-isolated strains against the corresponding original strains.

### 2.2 Statistical analysis

#### 2.2.1 Necrosis

Data were analysed using R, version 3.5.1. (R Core Team, 2019). A generalized linear model (glm) was constructed to evaluate the fixed effects of inoculation method (control/fungal strain) under different water treatments (25 %, 65%, and 100 %) on necrosis length in tips. Initial fixed explanatory variables in the necrosis length model included water treatment, year of isolation, host, origin (trophic mode), and tip-length (end of the experiment) was set as a random factor in the model. Further one-way-ANOVA (aov) was conducted, and Tukey’s HSD *post hoc* test was used to examine the differences between groups. Differences were considered statistically significant if the *p*-value was below the threshold of 0.01. The necrosis caused by the strains (6073, 6075 and 6085) from the study by Bußkamp et al. (2021), representing the re-isolates of strains 2697, 2715 and 5747, were compared with the same strain (necrosis caused by re-isolate versus long storage) using one-way-ANOVA (aov), and Tukey’s HSD *post hoc* test. The p-value threshold of 0.01 was chosen to indicate statistical difference between necrosis lengths.

#### 2.2.2 Growth

A generalized linear model (glm) in R version 3.5.1 (R Core Team, 2019) was constructed to evaluate the fixed effects of water availability treatments on sapling growth (height_end_ − height_start_). Initial fixed explanatory variables in the seedling growth model included height at the start, water treatment group and inoculation group (strain ID/untreated/mock-control). In addition, non-treated saplings were included in this model. Further one-way-ANOVA (aov) was conducted, and Tukey’s HSD test was used to examine the differences in growth (height_start_, height_end_, total growth) between water treatments.

## 3 Results

### 3.1 Pre-experiment detection of endophytes

Within three weeks, a total of 564 outgrowing mycelia from 414 incubated pine tissue samples (sapling 1: 87 samples, sapling 2: 108, sapling 3: 57, sapling 4: 84, and sapling 5: 78) were recorded. These mycelia, excluding four fungal species, could be assigned to 22 morphotypes including species of *Alternaria* (Frequency: 28.5%), *Diaporthe* (2.3%), *Coniochaeta* (0.2%), *Didymella* (0.4%), *Fusarium* (2.8%), and *Sordaria* among others, as well as *Cyclaneusma minus* (Butin) DiCosmo, Peredo & Minter (0.9%) and *Sydowia polyspora* (Bref. & Tavel) E. Müll. (4,1%)*, Microsphaeropsis olivacea* (Bonord.) Höhn. 1917 (17.0%) (Please find List of Taxa in **Supplementary Material**). *Diplodia sapinea* could not be isolated from any incubated tissue samples. The most abundant species with reference to frequency and continuity was a not further determined Didymellaceae (frequency: 37.9 %/continuity: 100%) followed by *M. olivacea* (continuity: 100%), *Alternaria alternata* (14.4%/100 %), *Alternaria* sp. 1 (6.7%/100%), *Alternaria* sp. 2 (5.7%/40%), and *D. polyspora* (continuity: 60%). All other species were isolated with less frequency.

### 3.2 Necrosis

All shoots inoculated with *D. sapinea* showed symptoms of infection: browning of the shoots and death of the needles (**Figure 1B**). All control plants remained healthy and symptomless. Some of the shoots treated with MYP showed minor discoloration of the phloem (0.6 cm arithmetic mean length) at the position where the shoot tip was excised. The necrosis length model (GLM) showed that necrosis size (length) was statistically affected by water availability, year of isolation, host and the trophic mode (origin of the isolate) (**Table 2** and **Figures 1C**, **D**).

**Table 2:** Statistical analysis of the necrosis length between the different water treatments, year of isolation, the different hosts, host origin and shoot length.

Variable	Fixed effect	Std. Error	t value	Sig.
Necrosis length	Water treatment	0.003	-10.082	< 2.2^e-16^
	Year of the isolation	0.039	9.909	< 2.2^e-16^
	Host	0.3941	21.600	0.0009494
	Origin of isolate	0.405	16.253	< 2.2^e-16^
	Shoot length	0.016	0.105	0.91638

**Figure 2:**
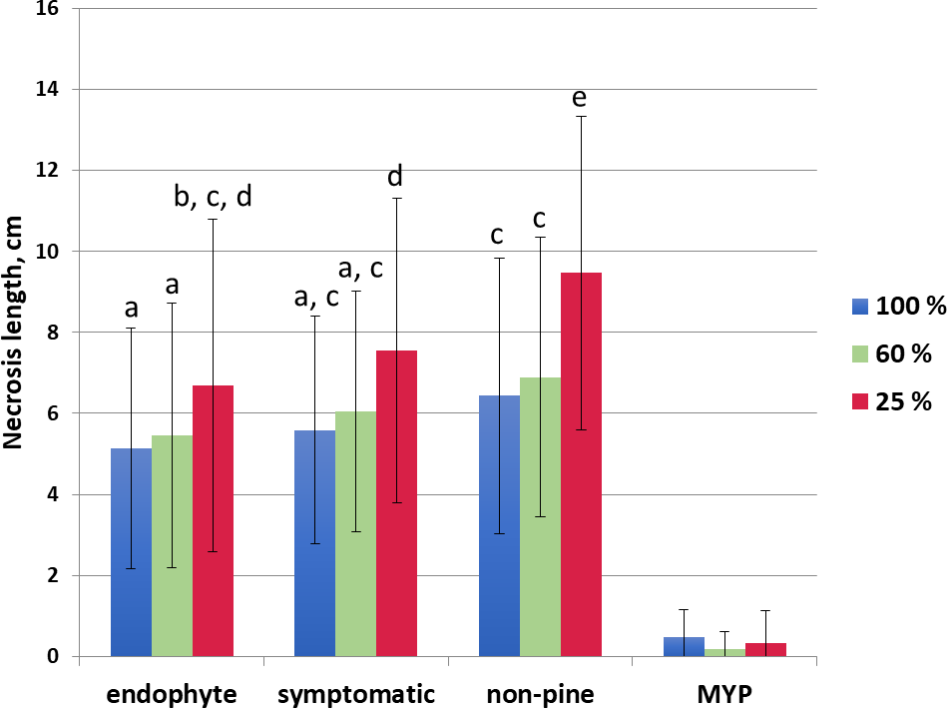
Necrosis length in cm of the side shoots for the different water treatments (100% blue, 60% orange, 25% red) grouped according to the *D. sapinea* strain’s origin (isolated from symptomless material = endophytic, from symptomatic material = symptomatic, isolated from non-pine hosts = non-pine and for the control the mock-treatment with MYP-agar plugs = MYP). The letters indicate the statistical differences.

The low water availability increased necrosis in each trophic mode (endophyte, symptomatic, non-pine), the greatest necrosis was observed in non-pine 25% water availability category (**Figure 2**). The necrosis length in the non-pine group was always statistically higher in each water availability class compared to the endophytic group (**Figure 2**). The necrosis length did not differ between the endophytic and the pathogenic (symptomatic) group (**Figure 2**).

### 3.3 Necrosis potential of isolates from re-infections

Strains NW-FVA 6073, 6075 and 6085 that originated from the study by Bußkamp et al. (2021) as re-isolates from (a) the originally endophytic strains NW-FVA 2697 and 2715 and (b) the originally pathogenic strain NW-FVA 5747 did not show differences in necrosis length under normal (100%) water availability. However, strain NW-FVA 6073 was able to cause statistically longer necrosis with 60% (*p*<0.01) and 25% (*p*=0.0001) water availability when compared to NW-FVA 2697. Soil moisture was measured over the duration of the experiment to facilitate adjustment of the amounts according to the temperature changes over the season and to allow sufficient watering for the 100% water group. Two low points were reached after two very hot weeks and the water supplied was immediately adjusted (**Supplementary Material**).

### 3.4 Identification of re-isolated *D. sapinea* strains by vegetative compatibility tests

*Diplodia sapinea* was isolated from 80.3% of all studied trees (n = 66 pine saplings). All inoculated *D. sapinea* strains apart from NW-FVA 2364 and NW-FVA 2697 (both originally assigned as asymptomatic and endophytic) were detected. The accordance of 36 re-isolated *D. sapinea* strains with the originally inoculated strains was proven by vegetative compatibility tests showing compatibility in 91.7% of all crossings (n = 108 crossings, three per each re-isolated strain). No *Diplodia sapinea* was isolated from the untreated control trees, although it was detected in one of the three trees inoculated with a MYP-plug (mock-control).

### 3.5 Growth

**Figure 3:**
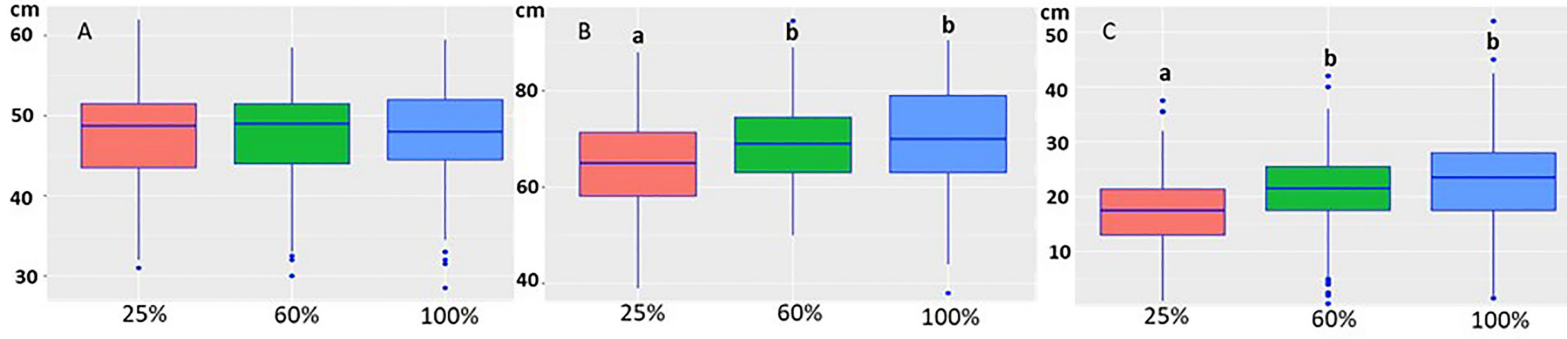
Boxplot of the height of the seedlings at the start of the experiment **(A)**, at the end of the experiment **(B)** under different water treatments (25%, 60% and 100%). The height of the seedlings was not statistically different at the start of the experiment in the different water treatments **(A)**. At the end of the experiment the height was statistically lower in the 25% treatment **(B)**. **(C)** The growth decrease was statistically significant in the 25% water group compared to 60% and 100% treatments. The letters indicate the statistical differences.

The growth model showed that water treatment (*p*= 5.74e-14) was the factor that affected growth (**Figure 3**). The starting height of the seedlings was not statistically different when they were randomly sorted into the water groups (**Figure 3A**). The 25% water treatment, representing drought conditions, adversely affected seedling growth, causing a statistically significant decrease when compared to the 60% and 100% treatments (**Figures 3B**, **C**). Neither height at the start (*p*=0.916) nor the isolate/inoculation method (*p*= 0.0967) affected growth (height_end_ − height_start_).

## 4 Discussion

Diplodia tip blight is an emerging threat to conifers worldwide due to the latent endophytic nature of its causal agent, *D. sapinea*, which alters its life-style under certain host-stressing conditions, such as drought (Bußkamp, 2018; Blumenstein et al., 2021b; Terhonen et al., 2021). While the fungus is favoured by warmer climate, it is spreading unnoticed to new areas (e.g. Brodde et al., 2019; Adamson et al., 2021; Terhonen et al., 2021), promoted by climate change. This situation also increases the incidence of this fungus infecting hosts other than the conifer trees it commonly infects (Langer and Bußkamp, 2021). In order to gain more insights into the possible impact of such host-passage and especially with regard to the increased likelihood of this in the future, and in relation to other host-pathogen interactions, we compared how different water treatments may affect the virulence and necrosis development of *D. sapinea* strains. The strains investigated were isolated from tissues of different health status and, thus, they were assumed to be in an endophytic or a pathogenic life-style; strains were also obtained from symptomatic tissues of non-pine hosts. Our results showed that, as expected, the Scots pine under drought stress had higher necrosis compared to the saplings that received an optimal amount of water. This result itself has been investigated in previous studies (e.g. Blumenstein et al., 2021b). The novelty of this study was the inclusion of re-isolated strains from a previous study and isolates from non-pine hosts. Interestingly, but also of concern, was that those infections led to the highest necrosis rates, independent of the water availability.

Necrosis length could be influenced by culture age and thus the vitality of the inoculated strains, and there could be loss of virulence of *D. sapinea* during storage under artificial conditions. We found that only one endophytic strain was able to cause longer necrosis compared to its original store strain. Further, the longer necrosis was observed only at lower water availabilities. It appears that the re-isolate was benefitting from the host stress. We used three re-isolates from previous research, but comparing more strains would yield more reliable results. However, based on these observations, the storage of *D. sapinea* does not alter the virulence of this fungus and the older strains can be used in inoculation studies.

The results of the pre-experiment showed that there were no endophytic *D. sapinea* strains associated with the three-year-old pine saplings, which fits the results of Blumenstein et al. (2021b). In that study, 18 different endophytic fungal species were isolated, which corresponds to the 22 morphotypes isolated in this study. In both studies, species of *Alternaria*, *Diaporthe, Didymellaceae, Epiccocum* as well as *M. olivacea* and *D. polyspora* were isolated. Additionally, in both trials the most common isolated species belong to the Didymellaceae. A possible explanation for the isolation of *D. sapinea* from a mock-control plant in the post-experiment isolation could be an infection of the single plant with *D. sapinea* during the experiment due to spores being present in the air in the greenhouse.

The statistical analysis showed that the different lifestyles (endophytic and pathogenic) do not yield a significant difference as we were able to demonstrate in our previous study (Bußkamp et al., 2021). This is most likely due to the timing of the experiments. In this study the highest temperature recorded was 32°C, whilst in the study by Bußkamp et al. (2021) it was 24°C. The optimal temperature for the growth of *D. sapinea in vitro* is 25–30°C (Milijasevic, 2006; Bußkamp, 2018), indicating that all the strains, regardless of their origin, preferred warm conditions. This means that the *D. sapinea* strains examined have the same pathogenic potential regardless of whether they were previously endophytic or virulent in the tree. Similar results were also obtained in studies by Flowers et al. (2001) and Stanosz et al. (2007).

**Figure 4:**
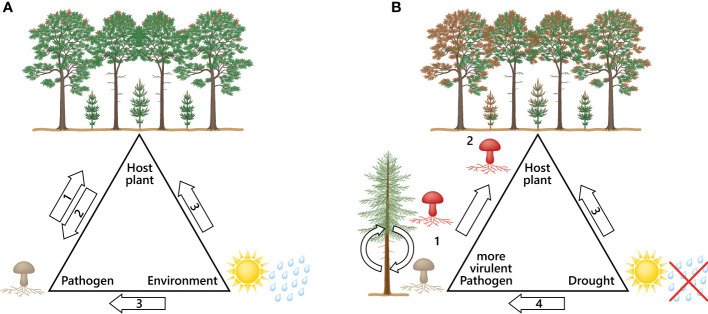
Disease triangles; **(A)** The disease triangle describes the factors involved for a disease to appear: (1) The virulence, distribution and infection capacity of a pathogen; (2) Host immune system; (3) suitable environmental conditions that benefit the pathogen and cause stress to the host. **(B)** Disease triangle with a scenario leading to severe outbreaks of pathogens: (1) Pathogen settles to a new host (host-jump; putative activation of more pathogenicity genes) leading to a more aggressive pathogen; (2) More aggressive pathogen can now infect its typical host with higher virulence leading to more devastating symptoms; (3) Drought weakens the host tree and makes it even more susceptible to pathogens; (4) Environmental change can benefit emerging and invasive pathogens leading to new host species and evolution of more aggressive strains.

The *D. sapinea* strains from the non-pine hosts were clearly the most aggressive, indicating a higher virulence compared to the strains originating from pine-hosts. The results of this study showed an even higher potential to cause more necrosis than in Bußkamp et al. (2021). A study by Ma et al. (2010) showed that horizontal gene transfer between different strains can change non-pathogenic to pathogenic strains, while other pathogenicity genes are described to be host plant dependent (Van De Wouw and Howlett, 2011). Infection studies with mutated plant pathogens showed reduced symptoms instead of none as described in the review from Van De Wouw and Howlett (2011), who studied fungal virulence factors. The results elucidate the complex role of pathogenicity genes inside their host, which indicates that genes involved in pathogenicity still require more in-depth investigation. The infection process is different for each pathogen-host interaction (Van De Wouw and Howlett, 2011). It remains to be investigated what causes such pathways to be activated in certain hosts, why they are not activated in others and why an organism may become more virulent after having gone through a “host-switch-passage”, as we observed in this study. Certain factors add to fungal virulence when interacting with different hosts (Urban et al., 2015; Shang et al., 2016). It may be assumed that the atypical host (here non-pine) activates pathogenicity genes that the common host usually does not trigger in that strength (**Figure 4**).

We urgently need research and new understanding of the interactions between trees and fungi within the continuum of climate change. The emergence of alien species alone due to climate change is not the only threat to the health of our forests (Prospero et al., 2021). The unseen fungal species (endophytes) that are normally in balance with the host tree may become opportunistic pathogens as the climate becomes more favourable to them. Drought causes strong abiotic stress for the host and disturbs plant growth (Szczepaniec and Finke, 2019). Similarly, low water availability has been found to reduce the growth of Norway spruce and increase the necrosis caused by fungal pathogens (Linnakoski et al., 2017; Terhonen et al., 2021). Here we show that all the isolated strains of *D. sapinea* benefit from the abiotic stress of the host. The pathogenicity (here defined as the length of visible necrosis) was boosted in each strain irrespective of the original mode of the fungi (asymptomatic or symptomatic/endophytic or pathogenic) as a result of drought. This is a text book example of a Disease Triangle (McNew, 1960), where a change in the environment increases the disease (**Figure 4**).

## Data availability statement

The datasets presented in this study can be found in online repositories. The names of the repository/repositories and accession number(s) can be found in the article/**Supplementary Material**.

## Author contributions

KB and JB planned and performed the greenhouse experiment. JB and GL performed the isolation of the endophytes, the necroses measurements, and the re-isolation of the fungi. ET contributed to the study design and analyzed the data. KB wrote the first draft. KB, JB, GL, and ET contributed to the final version of the manuscript. All authors contributed to the article and approved the submitted version.

## Acknowledgments

The authors are grateful for the great support from Annette Ihlemann and Martina Hille during the project and thank Etta Starick, Josephin Oelze, Ursula Rabel and Rebekka Schlößer for technical support and Steffen Bien for species confirmation (all NW-FVA). We also thank the students from University Göttingen Blessing Durodola, David Răscuţoi, Natalia Vasilevska, Adedolapo Akinbobola, Yasin Korkmaz and Anna Kolehmainen for their help with the laborious greenhouse work. We acknowledge support by the Open Access Publication Funds of the University of Göttingen. We thank Sees-editing Ltd for their English editing service of the manuscript.

## Conflict of interest

The authors declare that the research was conducted in the absence of any commercial or financial relationships that could be construed as a potential conflict of interest.

## Publisher’s note

All claims expressed in this article are solely those of the authors and do not necessarily represent those of their affiliated organizations, or those of the publisher, the editors and the reviewers. Any product that may be evaluated in this article, or claim that may be made by its manufacturer, is not guaranteed or endorsed by the publisher.

## Supplementary material

Supplementary Figure 1 The soil moisture data measured (60 trees) over the duration of the experiment for the three different water treatments (100% blue, 60% orange, 25% red).
