# Supplementary figures and images for: Diplodia tip blight pathogen’s virulence empowered through host switch
Source: Front Fungal Biol. 2022 Jul 28;3:939007. doi: 10.3389/ffunb.2022.939007 (PMC10512300; doi:10.3389/ffunb.2022.939007)

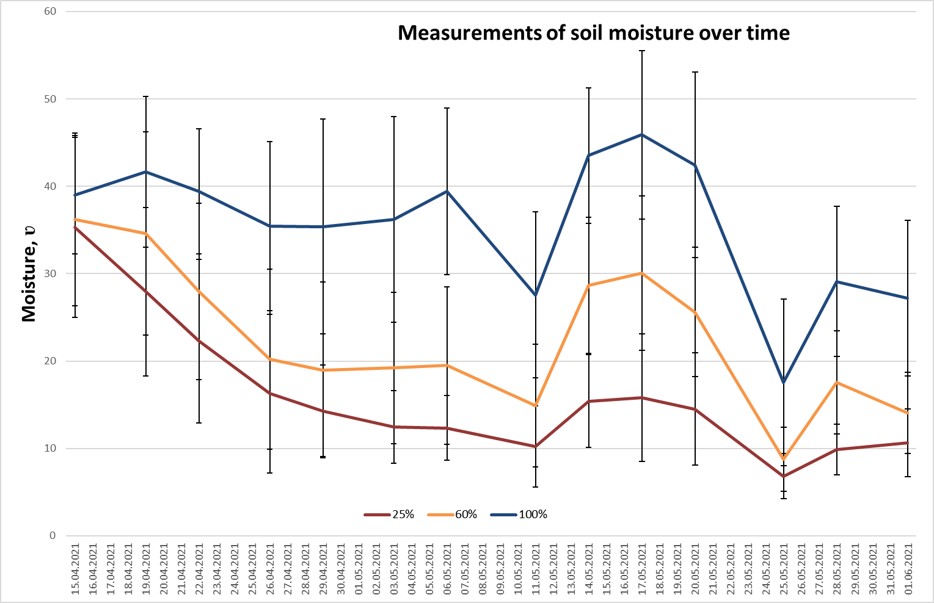

Supplement: Supplementary Figure 1 — The soil moisture data measured (60 trees) over the duration of the experiment for the three different water treatments (100% blue, 60% orange, 25% red). [file Image_1.jpeg]
